# Helicobacter cinaedi infection in patients with diabetes: a case report

**DOI:** 10.1186/s40064-015-0863-4

**Published:** 2015-02-10

**Authors:** Ryoichi Ishibashi, Susumu Nakamura, Minoru Takemoto, Chiaki Mukai, Koutaro Yokote

**Affiliations:** Department of Clinical Cell Biology and Medicine, Chiba University Graduate School of Medicine, Japan 1-8-1 Inohana, Chuo-ku, Chiba-shi, Chiba, 260-8670 Japan; Division of Diabetes, Metabolism and Endocrinology, Chiba University Hospital, Chiba, Japan; Kimitsu Chuo Hospital, Chiba, Japan

**Keywords:** Helicobacter cinaedi, Bacteremia, Diabetes

## Abstract

**Background:**

*Helicobacter cinaedi* causes bacteremia without characteristic clinical symptoms and is firstly isolated from human immunodeficiency virus (HIV)-positive homosexual men.

**Findings:**

Here we describe, for the first time case report, two female patients with diabetes who had *H. cinaedi* bacteremia. Some cases of *H. cinaedi* bacteremia may require long-term administration of multiple antibiotics prior to the resolution of infection.

**Conclusions:**

Therefore, these cases indicate that it is important to consider *H. cinaedi* in patients with diabetes presenting with bacteremia, especially in patients with poor glycemic control.

## Introduction

*Helicobacter cinaedi* is a Gram-negative enterohepatic bacterium of the Helicobacteraceae family that was first identified in 1985 among immunocompromised European and American men with human immunodeficiency virus (HIV) infections (Totten et al. 1985). In Japan, the isolation of *H. cinaedi* from blood culture was first reported in 2003 (Murakami et al. 2003), and it has since been occasionally isolated from samples obtained from patients with bacteremia and sepsis. In this report, we describe our experience with two patients with diabetes who had *H. cinaedi* bacteremia; in addition, we include a literature review.

## Case reports

Case 1: A 76-year-old female with a history of slowly progressive type 1 diabetes, treated for 30 years. She was being treated for rheumatoid arthritis (oral prednisolone, 5 mg/day, and had poor glycemic control specified by a glycated hemoglobin (HbA1c) level of 7.8% while on a regimen of 50% insulin lispro protamine suspension and 50% insulin lispro injection (HUMALOG® Mix50/50TM; Lilly USA, LLC, 2013) at 6 U in the morning and 4 U in the evening, as well as miglitol (10 mg/day) and mitiglinide (450 mg/day). She presented to the emergency unit of our hospital with a fever of 39°C, headache, erythema on the trunk, and joint pain. On admission, her body temperature was 40.2°C, pulse rate 134 beats/min, blood pressure 150/70 mmHg, height 145 cm, and body weight 38 kg. Routine laboratory testing showed an elevated leukocyte count of 11.8 × 10^3^ cells/μL and a C-reactive protein level of 19.21 mg/dL. Serological tests for syphilis and HIV were negative. As a result, we suspected a bacterial infection, especially because of the significant inflammation reaction in blood sampling; therefore, we decided to begin empiric cefepime therapy at 2 g/day. During hospitalization, our patient complained of a decreased appetite; thus, we commenced blood glucose monitoring on a sliding scale to maintain her preprandial blood glucose level at approximately 120 mg/dL. On day 2 of admission, her fever declined and erythema improved. On day 4, she was discharged after a change to oral cefotiam at 1800 mg/day. On day 9, she again developed high fever, poor appetite, and fatigue, and on day 10, she was readmitted to our hospital. On the same day, *H. cinaedi* was detected from a blood culture taken on the first day of admission; we accordingly started administering minocycline at 200 mg/day. A blood culture taken on the second day of re-admission was positive for *H. cinaedi*, indicating persistent bacteremia. However, because her general condition was improving, oral minocycline (200 mg/day) was continued. On day 18 after the initial admission, the third blood culture was taken, then she was discharged a second time. Later, we confirmed that her third blood culture was negative in an outpatient clinic and prescribed completion of the antibiotic regimen.

Case 2: A 47-year-old female diagnosed with type 2 diabetes 2 years before, and treatment started immediately thereafter. However, after a year, she stopped taking medications on her own decision. She presented to emergency department of our hospital with a fever of 38°C and bilateral lower leg pain for three days. She was hospitalized with a diagnosis of lower right leg cellulitis, and we began cefazolin administration at 3 g/day. On admission, her diabetic control was poor (HbA1c, 11.5%, postprandial blood glucose, 268 mg/dL); in addition, her body temperature was 39.2°C, pulse rate 106 beats/min, blood pressure 137/71 mmHg, height 166 cm, and body weight 61 kg. Routine laboratory testing showed an elevated leukocyte count of 13.8 × 10^3^ cells/μL and a C-reactive protein level of 4.49 mg/dL. Serological tests for syphilis and HIV were negative. Soon after starting antibiotic treatment, her fever subsided. Five days later, redness and swelling disappeared; therefore, we changed treatment to cefdinir at 900 mg/day. She was discharged on day 8. The following day, we confirmed *H. cinaedi* from the blood culture taken on day 1; nevertheless, we decided to adopt a wait-and-see approach because no exacerbation of disease was observed since her initial admission.

## Discussion

*H. cinaedi* is a rare infection primarily detected among HIV-positive patients in western countries. In Japan, most patients with *H. cinaedi* infection were HIV-negative, but immunocompromised (Araoka et al. 2014). *H. cinaedi* settles in the digestive tracts of humans, monkeys, dogs, and cats. Some cases of hospital-acquired outbreaks have also been reported (Rimbara et al. 2012). Neither of our patients had contact with animals, and because they came from different villages, they had no previous contact each other. Hence, the routes of infection remain unknown. Case 1 was prescribed 5 mg of prednisolone per day for rheumatoid arthritis, and both patients had poorly controlled diabetes. It has been reported that migratory, anti-infection, and phagocytic functions of leukocytes are compromised in patients with diabetes especially by high glucose levels. Therefore, patients with poorly controlled diabetes might be at a higher risk for *H. cinaedi* infection. However, there is no consensus on characteristic clinical symptoms of *H. cinaedi* infection and *H. cinaedi* is a fastidious organism, thereby making it difficult to clinically diagnosis.

The average separation and processing period of a blood culture to detect H. cinaedi is 5 days (Araoka et al. 2014), and the frequency of positive blood culture samples is reported to be 0.22% (Araoka et al. 2014). The actual frequency is probably higher because culturing this organism is difficult. Other reports have advocated genetic analyses, such as 16S-rRNA sequence and PCR-restriction fragment length polymorphism analyses (Oyama et al. 2012), for definitive diagnosis, although these methods are not feasible for routine laboratory analysis due to imbalance in cost–benefit ratio to detect very rare organisms. No guidelines have been provided for the treatment of *H. cinaedi* infections, even though this organism is reportedly sensitive to many antibiotics. In general, cephalosporins, carbapenems, tetracyclines, and aminoglycosides were found to be effective; however, various *H. cinaedi* isolates in Japan have shown increased resistance to penicillins and resistance to macrolides and quinolones (Kawamura et al. 2014). Therefore, some cases of *H. cinaedi* bacteremia may require long-term administration of multiple antibiotics prior to the resolution of infection.

In conclusion, two patients with poorly controlled diabetes presented with *H. cinaedi* bacteremia. Because *H. cinaedi* develops no characteristic clinical symptoms, it is important to consider *H. cinaedi* infection, especially in patients with poorly controlled diabetes who experience a relapse of infection after curative antibiotic treatment.

## Consent

Informed consent was obtained from all patients for being included in the study and for publication of this Case report.
